# Value of normative belief in intention to use workplace health promotion apps

**DOI:** 10.1186/s12911-022-01760-6

**Published:** 2022-02-02

**Authors:** Maren Junker, Markus Böhm, Mareike Franz, Tobias Fritsch, Helmut Krcmar

**Affiliations:** 1 Department of Informatics, Technical University of Munich, Boltzmannstraße 3, 85748 Garching bei München, Germany; 2 Department of Informatics, University of Applied Sciences Landshut, Am Lurzenhof 1, 84036 Landshut, Germany; 3 Mareike Franz, Munich, Germany; 4 Technical University Dresden, Dresden, Germany

**Keywords:** mHealth, Workplace health promotion, Structural equation modeling, Acceptance, Adoption

## Abstract

**Background:**

Mobile applications (apps) have started to be used for workplace health promotion (WHP). However, the factors that lead to the usage of apps in the workplace from the end-user perspective remain unclear.

**Methods:**

To investigate the research gap, the study evaluates a model for the adoption of WHP apps by combining the theory of planned behavior, the health belief model, and the technology acceptance model. A self-administered questionnaire with validated scales among 354 participants was used to evaluate the proposed model for WHP.

**Results:**

Although the study indicated a limited overall model fit, interesting aspects were derived. In particular, the study demonstrated that normative belief (especially), perceived usefulness, and attitudinal belief play important roles in the intention to use WHP apps.

**Conclusion:**

The study is among the first to validate the theoretical models of mHealth adoption for WHP. Moreover, it shows that not only normative belief but also adjustment to several target groups is a necessary factor to be considered in the development and implementation of an app for WHP.

## Background

Mobile technologies are currently used for various areas in the workplace. Apart from the general usage to support new ways of working [1] such methodologies may be further expanded toward the health of employees. As employers take an interest in supporting a healthy lifestyle among employees, the use of mobile applications (apps) for workplace health promotion (WHP) has been initiated [2]. The wide range of technical functions provides opportunities for integrating the dimensions of WHP, such as physical, psychological, and social health, into one program [3]. Therefore, this form of technology is expected to solve the currently low participation rates because apps have demonstrated high rates of usage and effectiveness in other areas of health [4]. Functions, such as automated feedback, social interaction, accessibility to health information, reminders and informational messages, and games are considered relevant for the health sector in providing interventions tailored to individual needs [5–7]. The advantages of technological interventions toward non-digital health promotion are further observed in the increased frequency of contacts with the program, possibility of adjusting the program to personal needs, the monitoring of health progress over a long time period and the possibility for health professionals to send personalized information [8]. Furthermore, apps cover daily activities independent of location and time [9]. From the employer perspective, apps may thus be an innovative means to provide WHP and reduce absenteeism (due to sickness) and presentism (being at work even if the employee is unwell, which leads to a high risk of long-term sickness later). Studies on mHealth for workplaces that aim to decrease the sedentary time of overweight individuals [10], improve physical activity [11], weight loss [12] or aim to reduce stress levels among middle managers [13] indicate the positive results of the mHealth apps. However, such studies mainly focus on effectiveness instead of acceptance in a practical setting. According to Keen [14], the technology itself does not hinder the implementation cycle, but the users who refuse certain technologies. Thus, research in this field should concentrate on adoption from the consumer perspective before implementing an app [7, 15, 16].

A study identified factors such as gender, health status, and age that influence participation in general workplace health interventions [17]. Furthermore, the app setup and content (e.g., competition, content adjusted to individual needs, or targeted communication) play a role in participation. However, whether the above mentioned reasons or other factors lead to the initial usage of health apps supported by mobile technologies (mHealth) for WHP remains unclear [18].

The study hypothesizes that acceptance factors may differ in WHP compared with privately used health apps due to the unique characteristics of the WHP setting. As stated by the varied literature on WHP, programs are often rejected because employees want to maintain a clear distinction between their private and workplace lives [17]. Furthermore, participants complain about initiatives that are not tailored to personal needs and preferences [19].

Additionally, factors of private health apps as stated in the literature may influence the intention to use a health app in a different manner in the workplace setting. For instance, trust in the developer is regarded and anticipated as an important factor for health apps usefulness [20]. Given the health technologies, perceived data security and privacy may play a role in mHealth acceptance, especially if data standards and regulations are haphazardly established [21]. For instance, if successes in behavior change, such as running distance, should be shared and posted in an app dashboard [22]. Such functionalities might be differently perceived if colleagues gain insight into these dashboards as a form of comparison with friends. This notion was supported by a study comparing privacy perceptions of individual data elements in private and workplace setting [23]. In this context, trust in the employer may be influential [24]. Additionally, although previous studies proposed that the community is in favor of the elements of gamification as competition and sharing of successes, this preference may pose pressure in the context of WHP. Furthermore, workplaces are unique in terms of people composition. Different people work together without personally opting for the social environment. Studies on mHealth have demonstrated that different social influences affect the intention to use an app [20], where the opinions of colleagues may differ from those of other individuals. Pressure from peers, colleagues, and supervisors may thus vary in another setting. Until now, less research focuses on the theoretical basis of the areas of technology usage for WHP as theories address health behavior or technology but rarely both. Thus, research is necessary before implementing such a program among workplaces [9].

### Theoretical background

**Fig. 1:**
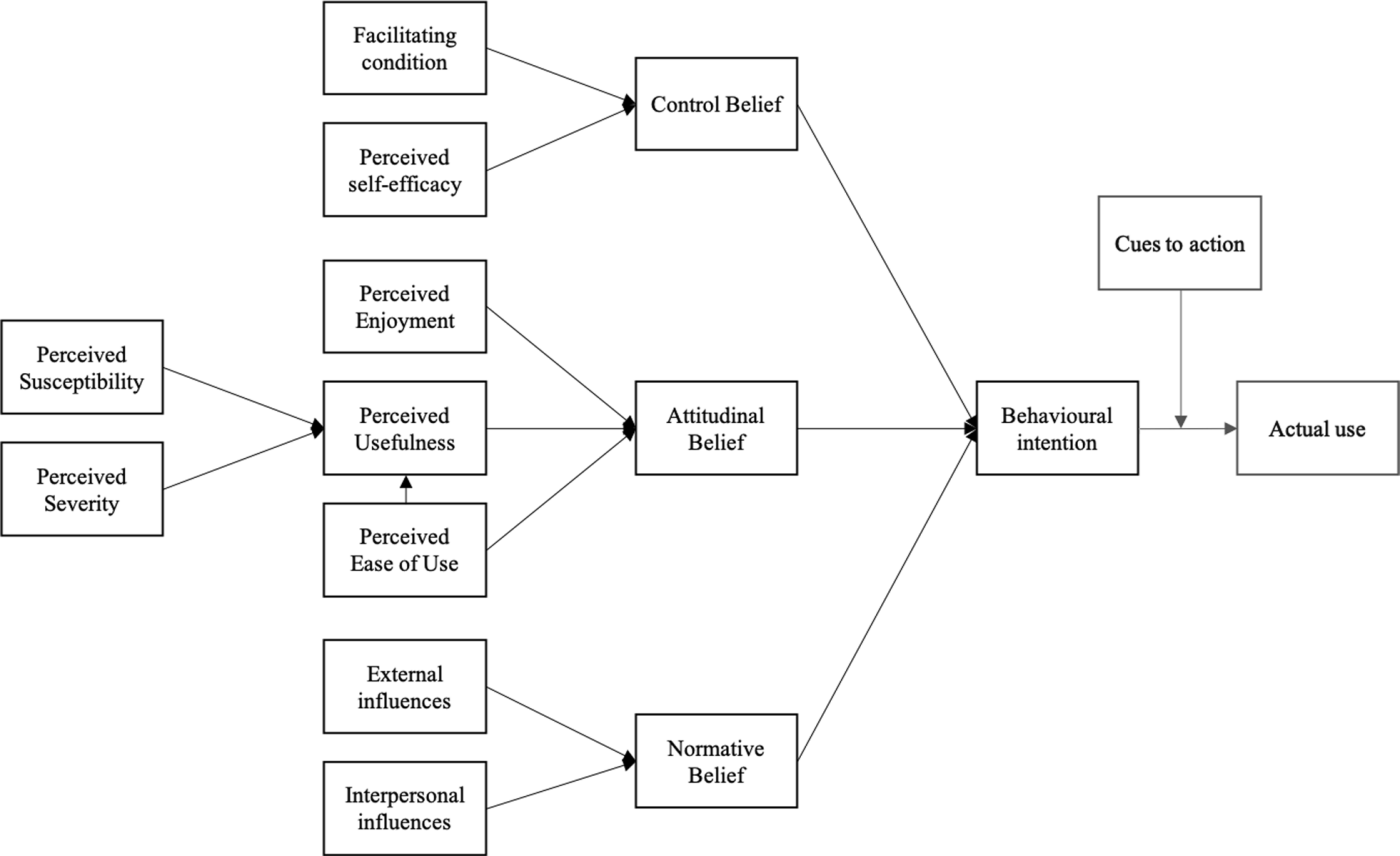
Tested model according to Melzner, Heinze and Fritsch 2014 [29]

Different theories, such as the theory of planned behavior (TPB), health belief model (HBM), social cognitive theory (SCT), transtheoretical model (TTM), Pender’s health promotion model, and self-determination theory (SDT), provide indications for behavior change and were applied to WHP, such as physical activity interventions [25]. However, their literature review proposed that none of the models cover participation in physical activities at the workplace in entirety. For the technical side of WHP apps, theories explaining the acceptance of technologies, such as the technology acceptance model (TAM) and unified theory of acceptance and use of technology (UTAUT), are considered frameworks for the research on the adoption of general apps. Comparing such theories for health intervention and behavioral change with those for technology acceptance, differences can be observed, which require consideration in the research on technically supported WHP interventions. Conclusively, whether a combination of the models can produce a model that is applicable to health technologies, such as mHealth, should be evaluated. Wu et al. [26] integrated the TAM and TPB for health professionals, which displayed a high predictive power for the adoption of apps in a hospital setting. Kim and Park (2012) developed the health information technology acceptance model (HITAM) for health technology acceptance based on the TAM [27], which Anderson et al. used for mHealth [28]. Additionally, Sari developed a model that integrates several aspects, such as performance expectancy, effort expectancy, social influence, facilitating condition, hedonic motivation, and health-related determinants from the UTAUT2 with the addition of privacy risk and personal innovativeness to test the applicability of the model in the workplace. Furthermore, demographic factors were tested for moderating effects [16]. Apart from Sari, Melzner et al. [29] developed a model for WHP via apps. The model is based on three validated models, namely, TPB, TAM, and HBM (Fig. 1).

Various studies provided support for the proposed interactions. For example, Wu et al. [26] found that attitude, control belief, and subjective norm exerted a significant influence on the behavioral intention to use a hospital app. Cocosila [30] exemplified the influence of individual perception in the adoption process by suggesting that perceived usefulness and ease of use are involved in the adoption process as proposed by the TAM in the context of information technology and health promotion. Additionally, internal and external influences are considered influential among WHP [31]. Brug [32] demonstrated that apps might lead to an increase in perceived behavioral control among participants because awareness of health behavior is increased through the visualization of food intake and exercise. Perceived behavioral control and self-efficacy further lead to a high levels of perceived ability to execute or intention to execute a certain behavior. In terms of health behavior, Campbell and Bopp [33] demonstrated that the interpersonal influences of colleagues and private environment are crucial for executing healthy behavior. As the proposed model of Melzner et al. [29] is one of the first models to be applied on apps in the workplace and is based not only on technology acceptance models, but also health behavior theories, their proposed model would be used to test its applicability to answer the following research question: Which factors of the proposed model significantly influence behavioral intention to use WHP apps?

## Methods

In order to answer the research question, a cross-sectional study was executed based on a German online self-administered survey that includes a short video about a WHP app in German language. Data analysis was done using structural equation modeling to validate the proposed theoretical model.

Before starting the actual questionnaire, participants were informed about the study and questionnaire structure. The questionnaire started with a short video before participants were asked to answer questions on the constructs and demographics.

The questionnaire covered all predictors proposed in the model except for actual usage and cues to action. The two factors could not be measured because of the nature of our study (users did not actually use the app). The remaining constructs proposed in the model were measured using validated scales derived from renowned journals that were additionally tested in a pre-test sample. The stated products in the original scale were subsidized by WHP apps. All constructs, except for two (rated on a 5-point scale), were rated using a 7-point Likert scale as proposed by the original scales and journals (Table 1). All items were translated into German by two researchers. They translated the questionnaire into German and back into English to guarantee the validation of the items. Differences were negotiated and agreed on. All questions were treated as mandatory in the questionnaire tool to guarantee that all answers are provided (Survey questions are shown in Additional file 1).

Besides the described constructs, demographic characteristics of the participants were measured (e.g. age, gender, job characteristics).

As described above, a short video was used to provide participants with a common understanding of a potential workplace app. This video was shown prior to the described questions and scales. The aim was to guarantee a common understanding on WHP apps, because short interviews prior to the study revealed that the imagination of such a possible app differed among individuals. The app shown in the video included common features found in health and lifestyle apps. It comprised several functions, such as tracking and analyzing nutrition behavior (i.e., lunch menu), physical activity using GPS and a map. Additionally, physical exercises were provided. Furthermore, the app included functions to increase work-life balance by providing information about childcare in kindergarten. All functions were revealed in a gamification element (point-gathering system), with a competition among departments. After the video, several questions regarding the content of the video were asked to ensure that all participants reached the same understanding and watched the video carefully. Those were (1) In the app, participants collect points for healthy behavior (Yes / No) (2) I can use the app to measure the distance I have walked (Yes / No).

A pre-test of the study was conducted among 197 individuals and lead to a few adjustments in the formulation of the items. Recruitment was done via social media. For the actual study, the participants were recruited via an online portal until the end of 2015. The portal provides a pool of participants. The survey was executed in the online tool Unipark. The target group was identified by creating a group with only participants considered eligible according to the inclusion and exclusion criteria and the correct answers to the video questions. The inclusion criteria were as follows: (1) participants spoke German. (2) They provided correct answers to the two questions on video content to guarantee that they paid attention to the video. (3) They had work experience of at least 12 months. Participants without smartphones, working part-time, or holding more than one job were excluded. All this was meant to ensure that they are capable of evaluating the usability of WHP apps. These criteria led to a study group with 354 eligible participants.

In the study, the need for an ethical approval was waived, because participation in the study was voluntary, data was gathered anonymously via the online survey and included no risks for the participants. Of course, participants were informed about the aims and procedure of the study prior to participation and could stop participation any time in the study. Participants were informed that by participating in the study they gave consent to use their data for scientific purposes.

**Table 1:** Measured scales

Construct	N items	$\alpha$	Mean (SD)	Journal	Refs.
Attitudinal belief	4	0.93	5.02 (1.29)	Decision support systems	[35]
Perceived severity (5-point Likert Scale)	3	0.87	2.97 (0.98)	Journal of health communication	[36]
Perceived susceptibility (5-point Likert Scale)	3	0.81	4.20 (0.69)	Journal of health communication	[36]
Perceived ease of use	4	0.90	5.51 (1.20)	Decision support systems	[35]
Perceived enjoyment	3	0.90	4.60 (1.36)	Journal of applied social psychology	[37]
Perceived usefulness	4	0.94	4.83 (1.37)	Decision support systems	[35]
Normative belief	3	0.83	4.06 (1.29)	IEEE Transactions on systems, man, and cybernetics-Part A: systems and humans	[38]
External influences	3	0.81	4.10 (1.19)	IEEE Transactions on systems, man, and cybernetics-Part A: systems and humans	[38]
Interpersonal influences	3	0.90	3.82 (1.43)	IEEE Transactions on systems, man, and cybernetics-Part A: systems and humans	[38]
Control belief	2	0.79	4.93 (1.34)	IEEE Transactions on systems, man, and cybernetics-Part A: systems and humans	[38]
Perceived self-efficacy	5	0.94	5.98 (1.15)	Occupational Medicine	[39]
Facilitating conditions	4	0.74	5.65 (0.95)	MIS quarterly	[40]
Behavioural intention	4	0.97	4.11 (1.67)	Electronic Commerce research and applications	[41]

### Statistical analysis

Data were analyzed using SPSS 22.0 and IBM SPSS AMOS 25. The scales were tested for reliability (Cronbach’s alpha), linearity, homoskedasticity, normal distribution, convergent and discriminant validity (with a threshold of 0.7 as recommended by Nunnally et al. [34]), common method bias, outliers, co-linearity, and correlations. Additionally, factor loadings using confirmatory factor analysis (CFA) were evaluated. Based on reliability, one item from the scale for facilitating conditions was omitted. Based on explanatory factor analysis, one item was omitted from the scale that measured external influences and one from control belief. After these tests, weaknesses were still found among facilitating conditions and control beliefs even after making adjustments. The reason for this notion is that the constructs loaded on similar factors, which may have caused decreased discriminant validity. Nevertheless, abnormality was not observed for all other tests. All prerequisites for the structural equation model were provided. The structural equation model was conducted in SPSS AMOS. The thresholds for model fit were applied according to Hu and Bentler [42].

## Results

In total, 565 participants completed the survey. Out of them, only 422 provided correct answers to questions related to video content and thus have paid attention to the video. Out of 422 remaining participants, 421 owned smartphones, whereas 400 had at least 1 year of work experience. Furthermore, 370 worked full time, and another 16 were excluded for holding several jobs. In total, only 354 participants were considered to meet the inclusion and exclusion criteria. The majority of the participants were male (63.8%) and aged between 21 and 30 years (48%) followed by 31 to 40 years (26.6%) and 41 to 50 years (18.1%). A high percentage of participants held a job experience of more than 10 years (47.5%) and did not work by shifts (65.8%) (Table 2). Additionally, 42.7%, 33.3%, and 24% considered their job not physically demanding, moderately physically demanding and physically/very physically demanding, respectively. The majority rated their health status as good or very good (73.7%). Specifically, 13.7% of the participants indicated being nearly completely inactive during leisure time, whereas 47.6% reported regular vigorous physical activity or workout at least 2 to 3 h per week. However, the majority of the respondents worked almost exclusively in a sedentary manner (28.2%) or sat and stood predominantly during work hours (35.9%). Experiences with health apps were mixed, where nearly 34% had never used a health app previously. This result indicates different knowledge bases and experiences among the technologies, thus representing a common workforce with a currently high average age. Regarding the outcome measure, the participants reported an average behavioral intention of 4.11 (on a scale ranging from 1 to 7 with high values indicating high levels of behavioral intention, SD = 1.67).

**Table 2:** Demographic characteristics

Characteristics	n	%
Sex		
Female	128	36.2
Male	226	63.8
Age		
Younger than 20 years	10	2.8
21–30 years	170	48.0
31–40 years	94	26.6
41–50 years	64	18.1
51–60 years	15	42.0
61 years or older	1	0.3
Health status		
Excellent	19	5.4
Very good	93	26.3
Good	149	42.1
Fair	83	23.4
Poor	10	2.8
Health app experience		
Uses health apps usually	95	26.8
Used health apps before, but stopped usage	139	39.3
Never used a health app	120	33.9
Physical activity level during leisure time N = 315 (definitions given in the Additional file 1)		
Physically inactive	43	13.7
Some light physical activity	121	38.4
Regular physical activity and training	127	40.3
Regular heavy physical training as competition	24	7.6
Regular working hours		
20–30 hours per week	8	2.2
31–40 hours per week	207	58.5
41 or more hours per week	139	39.3
Physical activity during work		
I almost only sit	100	28.2
Is it and stand mainly, I walk now and then	127	35.9
I walk mostly and move some material	78	22.0
I do heavy manual /physical work	49	13.8
Work industry		
Economy and finance	30	8.5
Service and trade	116	32.8
Health	32	9.0
Education and consulting	20	5.6
Industry and craft / trade	75	21.2
Gastronomy	12	3.4
Other	69	19.5
Job experience		
Between 1 and 3 years	63	17.8
Between 4 and 10 years	123	34.7
More than 10 years	168	47.5

### Structural equation modelling

Structural equation modeling was used to validate the proposed theoretical model [29]. The result revealed the critical model fit parameters as follows: normed fit index (NFI) =.78, comparative fit index (CFI) =.829, and root mean square error of approximation (RMSEA) =.087. In terms of squared multiple correlations, the study demonstrated that, in total, only 52.9% of the variance in behavioral intention can be explained using the proposed model (Table 3). Although the variance explained is considered small [43], only a few studies using structural equation modeling on mHealth exists and illustrate high model fit values. For instance, a study that used the UTAUT2 among college-aged respondents for mHealth indicated that 63% variance could be explained [44]. Dou et al. [45] conducted a study on apps for chronic disease management using the HBM and TAM (i.e., measuring resistance to change, perceived usefulness, perceived ease of use, perceived health threat, relationship with the doctor, self-efficacy, and usage experience). However, only 41.2% of variance could be explained.

Evidently, the highest variance can be explained in normative belief at 63.9% mostly by interpersonal influences ($\beta$ =.758). Two estimated paths did not reach statistical significance (i.e., perceived ease of use attitudinal belief and facilitating conditions control belief). In addition, several estimations were derived with a poor effect size of $\beta$ less than .2.

**Table 3:** Squared Multiple Correlations

Construct	Estimate
Perceived usefulness	0.377
Control belief	0.358
Attitudinal belief	0.549
Normative belief	0.639
Behavioral intention	0.529

**Fig. 2:**
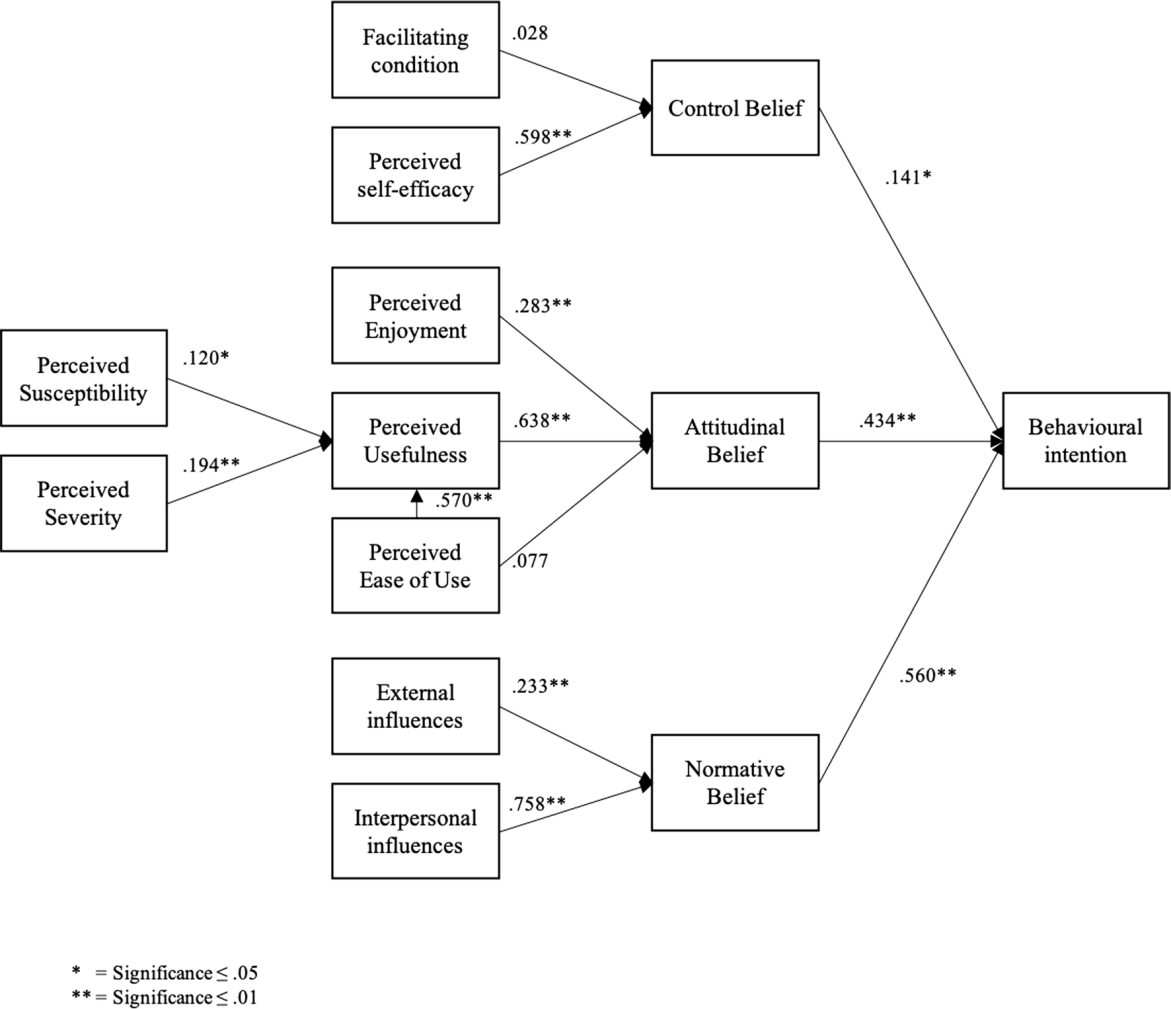
Standardized regression weights and signicance of paths

Figure 2 presents the regression weights, which revealed that the level of association between normative belief and behavioral intention is higher than that for control belief and attitudinal belief. Control belief is less associated with behavioral intention. When considering the factors associated with the three main constructs, the study observed interesting results. Within the minor role of control belief, facilitating condition is non-significantly related to control belief. This finding may be explained by the difficult measurements and poor associations. Moreover, ease of use is non-significantly associated with attitudinal belief. The construct is associated with usefulness only. Furthermore, perceived susceptibility and severity only explain a small proportion of the variance of usefulness. However, behavioral intention seems to differ among healthy and less healthy individuals. Interestingly, enjoyment is less associated with attitudinal belief compared with usefulness, which includes that rational thoughts seem to overload enjoyment. Lastly, given normative belief, social relations (interpersonal influences) seem to play a major role in acceptance and should be considered when implementing such an app. Afterward, perceived susceptibility and severity were tested as moderating variables between perceived usefulness and attitudinal belief. However, no significant correlations were revealed.

### Group differences

Independent t-tests for behavioral intention were conducted for several groups to examine acceptance among different groups. Group differences were tested for age, gender, health app experience, innovativeness, and health status. All factors except for age demonstrated significant differences in intention to use a WHP app (Table 4). Therefore, the study inferred that a workplace health app could be used for different age groups. However, various factors should be considered, whereas options of personalization may be necessary. For instance, innovativeness and use of a previous health app support usage. Furthermore, individuals with a positive perceived health status feel less need to use such an app. Interestingly, women are more motivated to use the app than men are. Thus, the study proposed that the influence of previous experience, gender, health status and innovativeness should be added to future models. This finding is in line with TAMs, such as the UTAUT.

**Table 4:** Group differences for intention to use a WHP app

Construct	Group 1	Group 2	Average difference (Group 1–Group 2)	*p* value
Health status	Health perceived as “excellent”, “very good” or “good”	Health perceived as “fair” or “poor”	− 0.41	0.045
Health app usage	Have used a health app or uses it on a regular basis	Have never used a health app	0.67	0.00
Age	≤ 30 years	≥ 31 years	0.04	0.83
Age	≤ 40 years	≥ 41 years	− 0.03	0.89
Innovativeness	< average value $(< 4.63)$	> average value $(> 4,63)$	− 0.93	0.00
Gender	Female	Male	0.40	0.03

## Discussion

WHP apps are deemed a big field of interest in the future. However, theoretical knowledge on acceptance of WHP apps is scarce [46]. Melzner et al. [29] were among the first to develop a model for explaining the adoption of mHealth for WHP using HBM, TAM, and TPB. In this light, the current study tested the model for usability and validity. The study aimed to identify the factors that may facilitate or hinder the usage of such apps in the future as well as to produce guidelines for the development of such apps. As previously mentioned, the model only explained 52.9% of variance and thus did not reveal sufficient values for model fit and R2 values for behavioral intention as suggested by Hooper et al [43]. Nevertheless, the variance explained seem to be average compared with other studies on health apps.

Moreover, the study noted several interesting results and drew interpretations considering the analysis of the proposed model. Evidently, the workplace with its unique characteristics triggers different acceptance factors compared with private health apps. As previously described, one unique characteristic is the composition of individuals as health promotion programs can reach diverse people in the workplace [47]. Additionally, the influence of the direct social environment differs from those of the social environment and in private life due to the potential hierarchal structure. The results reflect this notion, which indicates that normative belief is an important factor when considering WHP apps and demonstrates a certain extent of pressure derived from the private or professional environment. A higher level of association is observed between normative belief and behavioral intention than between normative belief and attitudinal belief. External influences, such as those from the public or experts, are less influential. This finding seems to contrary to other settings. For instance, Yuan et al. [44] tested the UTAUT on health and fitness apps and illustrated that performance expectancy, hedonic motivation, price value, and habit significantly predicted the users’ intent of continued usage. Other factors, such as effort expectancy, social influence, and facilitating conditions were non-significantly related to the intention of continued usage of health and fitness apps. Additionally, Beldad and Hegner [20] used two categories of normative belief or social norm, namely, injunctive and descriptive, where injunctive belief is expected to be closely related to the social norm provided in the theory of reasoned action. The authors tested the following factors: perceived ease of use, perceived usefulness, injunctive social norm, descriptive social norm, trust in the app developer, and health valuation on the intention to use a health app. The results demonstrated that only perceived ease of use ($\beta$ =.43), perceived usefulness ($\beta$ =.31), and injunctive social norm ($\beta$ =.14) exerted a significant direct effect on intention to use [20]. However, such associations were lower compared with those in the current study. In summary, the social influences of apps for WHP differ from those of private health apps. Furthermore, the study assumes that pressure from the work environment exerts a great influence on the intention to use an app. These results reveal that employees need to feel supported by the work-related social environment to encourage the use of the application for health improvement. For example, group exercises in apps may enhance competition among colleagues. Furthermore, support can be increased by integrating managers into the program, such as by allowing participation within work time [46, 48]. Interestingly, Beldad and Hegner [20] also showed that perceived ease of use is considered more important than perceived usefulness. In the current study, ease of use significantly influenced usefulness, but did not directly influence the attitude toward the app. Accordingly, the rational considerations of users seem more essential, even more than perceived enjoyment. Therefore, the authors hypothesize that the benefit of such an app is more important than leisure. The need for a perceived benefit compared with other private apps is further supported by qualitative research in this field [49]. However, other studies reinforced the importance of perceived enjoyment by analyzing the concept of hedonic motivation using UTUAT2. Thus, the underlying concepts of psychological need fulfillment can be expected to explain this importance [50]. These concepts have not been validated in the workplace setting but may further influence behavior intention and app usage in terms of job-related need fulfillment. Given the factors severity and susceptibility, Beldad and Hegner [20] revealed that no significant influence exists between health valuation and perceived usefulness, whereas McGloin [51] provided support for the path between health consciousness and perceived usefulness.

The hypotheses in the literature stated that individuals who are more engaged in their health may have already defined their programs and methods for health improvement and show less interest in such an app. This notion supports the literature that revealed that employees frequently prefer their own health activities instead of WHP [19]. Interestingly, control belief, self-efficacy, and facilitating condition to use such an app are less relevant than that expected by Melzner et al. [29]. Apart from issues regarding the scales used in the present study, one probable explanation is that employees expect to obtain support from the employers regarding their usage, which renders social pressure and attitude toward the technology as more important factors. Interestingly, in the health care context, facilitating conditions as a support were considered as one of the most important aspects [52]. Such results reveal the relevance of social environment and inclusion of different stakeholders in the implementation process of such an app. Upon comparing the results of the present study with those of studies conducted in a private setting, this finding demonstrates the advantages of such an app compared with ordinary health promotion because individuals who are less interested in health promotion in general may become engaged through positive social pressure. Evidently, this scenario may include the risk of negative social pressure and perceived enforcement. Additionally, the results support the importance of marketing and demonstrations to convince employees about the individual usefulness of the app. When considering the results of the group differences on behavioral intention to use, a further conclusion can be drawn. The study reveals that the initiators of WHP apps should consider their target group and its needs and expectations to maximize individualization due to differences in demographic characteristics and behavioral intention. This result partially agrees with UTAUT2, which hypothesized that gender, age, and experience play a role in technology acceptance [53] as well as that proposed by Sari et al. [16]. For instance, individuals with less experience of health apps and low levels of technical innovativeness displayed low levels of intention to use such an app, which demonstrates that these individuals may require further assistance to overcome first-usage barriers. Interestingly, individuals with poor health were significantly more engaged with the app than those with better health status were. Apart from these interpretations from the results of the study, the overall value of the model in predicting usage leaves room for improvement. In other words, the proposed model does not seem to reflect all factors that influence the acceptance of WHP apps [29]. Thus, other factors may potentially significantly influence the decision or factors load differently than that proposed by the model. For instance, Kim and Park [27] integrate the factors health status and perceived threat into the HITAM, which is supported by our finding in the difference between health status and intention to use the WHP app. Furthermore, the authors added the factor HIT reliability, which includes output quality and result demonstrability. This construct may be extended by perceived security and privacy, which are expected to play a role in the future of mHealth [54]. Additionally, the two factors found to influence the intention to use the WHP app, namely, gender and experience, are supported by the UTAUT2 and may require further consideration in the future model of the WHP app. The UTAUT further reinforced the importance of not only hedonic motivation but also effort and performance expectancies, which may need a detailed evaluation in the future for WHP apps. This also relates to experience with technology and may even be influenced by the industry people work in e.g. IT experts may have more technical experience. Therefore, additional factors and demographics would need to be evaluated in detail in the future. Lastly, the model would also need to evaluated in the practical setting in order to test validity among actual usage and not only intention to use as argued by several authors.

### Limitations and future research

The different limitations of the study should be mentioned. First, the participants were recruited using an online platform, which may introduce selection bias by attracting people with a general interest in health and/or technology. Differences among participants may have occurred in terms of experience in previous WHP means and work environment, such as company size and culture. Furthermore, the app in the video and interpretation of items may have been differently perceived. In total, 143 participants were excluded as they failed to provide the correct answers to the questions about the app. Therefore, they may have a different understanding of the app. In terms of generalizability, the study was conducted among only German speaking participants. Also, the study measured only behavioral intention and ignored actual behavior due to the study design. Therefore, the association between behavioral intention and actual behavior, which is expected to be mediated by cues to action, has not been further evaluated. Further research is necessary in terms of actual behavior and cues to action. The literature mentioned perceived credibility and security as factors. However, the current study also overlooked these aspects. Furthermore, previous experiences in the use of health app play an important role and should be investigated in detail. The study further aimed to investigate the importance of normative beliefs. Therefore, specifying support groups, such as friends or colleagues, and their reachability should be considered. Lastly, the factors for individualization of the app might underlie the usefulness of the app and should be addressed in future research.

## Conclusion

This study provides different implications for research and practice. First, it highlights the research gap in terms of a theoretical grounding for the research on WHP apps. As many apps are developed without a validated theoretical background, theoretical grounding is necessary. It provides insights into the acceptance of such a technology in WHP and further development for other technologies. Additionally, it provides important information for employers who consider using apps as a new means for WHP. In a nutshell, the proposed model based on the TAM, TPB, and HBM indicates a low model fit. The study revealed that the unique characteristics of WHP influence the acceptance factors compared with private health apps. Thus, the study concluded that control belief and facilitating conditions are less important in the present context; but attention should be given to perceived usefulness, ease of use and, especially, normative belief, which was found to be the main difference among other settings. Considering the ideas of employees during the development of app content is crucial to address individual needs. In addition, management support is essential and should be stimulated. Further, the study elucidated that the communication strategy of companies is important for adjusting to the needs of employees and thus convincing them to participate. Moreover, the study inferred that apps can provide a solid foundation for future WHP as it can be tailored to different target groups in terms of age, gender, health status, and innovativeness. The study provides guidance for practical settings and app developers. Furthermore, researchers can base their work on the model as an extension of health theories and technology acceptance.

## Supplementary Information

**Additional file 1:** Survey questions.

## Data Availability

The datasets used and/or analyzed during the current study are available from the corresponding author on reasonable request.

## Abbreviations

HITAM: Health information technology acceptance model
HBM: Health belief model
SCT: Social cognitive theory
SDT: Self-determination theory
TAM: Technology acceptance model
TPB: Theory of planned behavior
TTM: Transtheoretical model
UTAUT: Unified theory of acceptance and use of technology
WHP: Workplace health promotion
