# Supplementary material for: Value of normative belief in intention to use workplace health promotion apps
Source: BMC Med Inform Decis Mak. 2022 Feb 2;22:30. doi: 10.1186/s12911-022-01760-6 (PMC8812227; doi:10.1186/s12911-022-01760-6)
Supplement: Supplementary file 1 — Additional file 1: Survey questions. [file 12911_2022_1760_MOESM1_ESM.pdf]

**Construct** *(Reference the scale was adapted from)*	**Measured items** (adapted from the references)	**Scale**
**Attitudinal beliefs** *(Cheng et. al., 2006 "attitude")*	I think that using the app is a good idea.<br>I think that using the app is pleasant.<br>In my view, using the app is a wise idea.<br>In my opinion, it is desirable to use the app	7-point Likert scale: Strongly agree - Strongly disagree
**Perceived susceptibility** *(Witte, Cameron, McKeon, Berkowith, 1996)*	I believe that physical or mental health difficulties are severe.<br>I believe that physical or mental health difficulties are serious.<br>I believe that physical or mental health difficulties are significant.	5-point Likert scale: strongly agree-strongly disagree
**Perceived severity** *(Witte, Cameron, McKeon, Berkowith, 1996)*	I am at risk for getting physical or mental health difficulties.<br>it is likely that i will contract physical or mental health difficulties.<br>It is possible that i will contract physical or mental health difficulties.	5-point Likert scale: strongly agree-strongly disagree
**Perceived usefulness** *(Cheng et al. 2006)*	Using the app would enable me to accomplish my tasks more quickly.<br>Using the app would make it easier for me to carry out my tasks<br>I would find the app useful.<br>Overall, I find using the app to be advantageous	7-point Likert scale: Strongly agree - Strongly disagree
**Perceived ease of use** *(Cheng et al. 2006)*	Using the app is easy for me.<br>I find my interaction with the app clear and understandable.<br>It is easy for me to become skillful in the use of the app.<br>Overall, find the use of the app easy.	7-point Likert scale: Strongly agree - Strongly disagree
**Perceived enjoyment** *(Davis et. al.,1992)*	I would find using the app to be enjoyable.<br>Using the app would be pleasant.<br>I would have fun using the app (likely/unlikely)	7-point Likert scale: Strongly agree - Strongly disagree
**Normative beliefs** *(Bhattacherjee, 2000)*	People (peers and experts) important to me would support my use of the app.<br>People who influence my behavior would want me to use the app instead of alternative means.<br>People whose opinions I value would prefer that I use the app.	7-point Likert scale: Strongly agree - Strongly disagree
**External influences** *(Bhattacherjee, 2000)*	Experts advise people to use apps for improving their health<br>I read/saw news reports that using mobile application is a good way to avoid physical and mental health difficulties.<br>The popular press depicts a positive sentiment for using mobile health applications.<br>Mass media reports influence me to try out mobile health applications.	7-point Likert scale: Strongly agree - Strongly disagree
**Interpersonal influences** *(Bhattacherjee, 2000)*	My peers/colleagues/friends would think that I should use mobile application for improving my health status.<br>People I know would think that using mobile application was a good idea.<br>People I know would influence me to try out mobile applications for improving my health status	7-point Likert scale: Strongly agree - Strongly disagree
**Control beliefs** *(Bhattacherjee, 2000)*	I would be able to use the app well for improving my health<br>Using the app is entirely within my control.	7-point Likert scale: Strongly agree - Strongly disagree

**Perceived self-efficacy** *(Jones 1986)*	I am fully capable of using the app.<br>I am confident in my ability to use the app.<br>Using the app is well within the scope of my abilities.<br>I do feel I am qualified for the task of using the app.<br>My past experience increases my confidence that I will be able to successfully use the app.	7-point Likert scale: Strongly agree - Strongly disagree
**Facilitating conditions** *(Venkatesh, Morris, Davis and Davis, 2003)*	I have the resources necessary to use the app<br>I have the knowledge necessary to use the app.<br>The system is compatible with other apps I use.<br>A specific person (or group) is available for assistance with app difficulties	7-point Likert scale: Strongly agree - Strongly disagree
**Behavioral intention** *(Suh & Han, 2003)*	I intend to continue using the app in the future.<br>I expect my use of the app to continue in the future.<br>I will frequently use the app in the future.<br>I will strongly recommend other to use this app.	7-point Likert scale: Strongly agree - Strongly disagree
**Smartphone Experience**	How long have you owned a smartphone?<br>a) For less than 1 year<br>b) For 1-2 years<br>c) Since 3- 4 years<br>d) For more than 4 years<br>e) I do not own a smartphone	
**Innovativeness**	When I hear about a new app, I look for a way to experiment with it<br>Compared to my friends/colleagues, I am always one of the first to try out a new app<br>In general, I am hesitant to try out new apps<br>I like to experiment with a new app	7-point Likert scale: Strongly agree - Strongly disagree
**Health Apps**	Have they ever used a health app (exercise, nutrition, information)?<br>a) Yes, I regularly use such an app<br>b) Yes, but I do not use it anymore<br>c) No, never	
**Health status**	I would say my health status is ...<br>a) excellent<br>b) very good<br>c) good<br>d) alright<br>e) bad	
**Demographic questions**	What is your gender?	Female/Male
	How old are you?<br>a) 20 years or younger<br>b) 21- 30 years<br>c) 31- 40 years<br>d) 41- 50 years<br>e) 51- 60 years<br>f) f61 years or older	
	What is your work situation? (multiple answers possible)<br>a) I work full time<br>b) I work part time<br>c) I am not working at the moment<br>d) I am a student/ pupil/ trainee	

	For how many years have you been employed? (Please subtract interruptions due to parental leave, unemployment, etc. from the time)<br>a) For less than 6 months<br>b) For 6-12 months<br>c) For 1-3 years<br>d) For 4-10 years<br>e) For more than 10 years	
	What industry are you working in?<br>a) Economy and finance<br>b) Service and trade<br>c) Health<br>d) Education and consulting<br>e) Industry and trade<br>f) Gastronomy<br>g) Other	
	How many hours do you work on average per week?<br>a) Less than 20 hours<br>b) 20- 30 hours<br>c) 31- 40 hours<br>d) 41 or more hours	
	Do you currently work regularly outside of regular working hours (7am- 8pm)?	Yes/No
	Do you currently perform two professions/ jobs?	Yes/No
**Physical Activity**	To what extent do you exercise while working?<br>a) I almost only sit<br>b) I sit and stand mainly. I walk now and then<br>c) I walk mostly, and move some material<br>d) I do heavy manual/physical work	
	How physically demanding do you find your work?<br>a) Very physically demanding<br>b) Physically demanding<br>c) Intermediate physically demanding<br>d) Not physically strenuous	
	How much exercise or physical activity do you do in your free time?<br>a) Physically inactive: I am almost completely inactive during leisure time: reading, watching TV, movies, using the computer, or activities you do while sitting<br>b) Some light physical activity: I perform physical activities of at least 4 hours per week such as bicycling or walking to work, walking with family, gardening, fishing, ping pong, bowling, etc.<br>c) Regular physical activity and training: I spend time doing heavy gardening, running, swimming, tennis, badminton, fitness, or similar activities of at least 2-3 hours per week<br>d) Regular heavy physical training as competition: I spend time racing, skiing, swimming, soccer, handball, etc. several times a week	

Supplementary file 1: Survey questions
